# Robust Pose and Inertial Parameter Estimation of an Unknown Aircraft Based on Variational Bayesian Dual Vector Quaternion Extended Kalman Filter

**DOI:** 10.3390/e28050549

**Published:** 2026-05-12

**Authors:** Shengli Xu, Yangwang Fang, Hanqiao Huang

**Affiliations:** Unmanned System Research Institute, Northwestern Polytechnical University, Xi’an 710072, China

**Keywords:** parameter estimation, dual quaternions, variational Bayesian, robust tracking

## Abstract

Accurately determining the parameters of an unmodeled spacecraft is crucial. Filtering methods that are resilient to uncertainty, employing dual quaternion frameworks to ascertain orientation and position, introduce a design for an extended Kalman filter based on variational Bayesian inference and dual vector quaternions (VB-DVQEKF) to carry out parameter estimation for a non-cooperative spacecraft. The system kinematics and dynamics are modeled using dual vector quaternions, rendering the representation manifestly concise. The method achieves thoroughness by accounting for the coupled interactions between translational and rotational motions. Furthermore, to address uncertainties in the measurements, a variational Bayesian approach is employed for the dependable simultaneous estimation of state parameters and measurement noise covariance. Mathematical simulations are used to verify the proposed VB-DVQEKF, and its robust capabilities are demonstrated through comparisons with several conventional parameter estimation techniques, including the conventional DVQ-EKF and the Sage–Husa adaptive DVQ-EKF (SH-DVQEKF). Quantitative results based on root-mean-square error (RMSE), convergence time, and final estimation error confirm that the proposed VB-DVQEKF achieves the smallest steady-state error among the compared methods and remains stable under white-burst, gradient (drift), and outlier-type measurement anomalies.

## 1. Introduction

Given that autonomous rendezvous and docking (AR&D) [1,2,3,4] is a necessity, missions for on-orbit servicing (OOS) have attracted considerable scholarly attention in recent years. With the increasing number of aging or malfunctioning spacecraft in orbit, OOS missions such as refueling, repair, debris removal, and component replacement have become strategically significant for extending satellite lifetimes and maintaining sustainable space operations. For these operations, it is critically important to precisely determine the state parameters of non-cooperative targets that are freely floating. Such targets typically lack cooperative markers, active beacons, or communication interfaces, which makes state acquisition highly challenging. The inherent tumbling motion of these targets further compounds the difficulty, as their rotational dynamics are often unknown, time-varying, and coupled with translational motion, thereby introducing significant nonlinearity and uncertainty into the estimation process.

Although vision-based sensors are widely employed to extract relative pose measurements [5,6,7], these methods suffer from notable limitations. Specifically, vision-based approaches primarily focus on extracting geometric pose information from image features, while neglecting the underlying kinematic and dynamic constraints of the target. As a consequence, they often incur high computational burdens due to intensive image processing and feature matching procedures. Moreover, they provide inadequate consideration of space environmental effects, such as illumination variation, occlusion, background interference, and sensor noise, all of which may deteriorate measurement reliability. More importantly, these methods are generally restricted to pose estimation and are unable to acquire parameters beyond relative position and attitude; inertial parameters, including mass properties and rotational inertia, remain unidentified. Such limitations significantly restrict their applicability in high-precision OOS missions that require comprehensive dynamic characterization of the target.

To address these deficiencies, researchers have proposed parameter estimation methods that integrate vision-based sensor measurements, target motion equations, and the Extended Kalman Filter (EKF). By embedding the physical motion constraints into the estimation framework, these approaches enhance estimation consistency and reduce reliance on purely geometric observations. In particular, by introducing dual quaternions to describe both rotational and translational motion within a unified mathematical framework, these methods consolidate the relevant parameters into a compact and coordinated expression. Dual quaternions offer the advantage of representing rigid body motion in a singular algebraic structure, thereby avoiding the singularities associated with Euler angles and the redundancy inherent in homogeneous transformation matrices.

Specifically, ref. [8] established an error dual quaternion kinematic model to characterize the deviation between estimated and true states, laying the foundation for filter-based estimation. Subsequently, refs. [9,10] developed pose estimation algorithms based on a dual quaternion kinematic model combined with the EKF, demonstrating improved performance in nonlinear estimation scenarios. However, such work primarily focuses on kinematic modeling, and dynamic models have not been adequately integrated into the target parameter estimation framework. The absence of dynamic constraints limits the estimator’s ability to capture inertial properties and dynamic coupling effects, thereby restricting overall estimation accuracy and robustness.

To bridge this gap, ref. [11] introduced Dual Vector Quaternions (DVQs) to develop models incorporating dynamic behavior. By reformulating the dual quaternion representation and emphasizing its vector components, DVQs provide a structured means to embed both kinematic and dynamic relations into the estimation process. Nevertheless, despite these advances, the aforementioned dual quaternion-based estimation methods still encounter performance bottlenecks when handling measurement uncertainty. In practical OOS scenarios, sensor noise characteristics may deviate from nominal assumptions, and uncertainty in measurement covariance can significantly degrade filter performance.

During OOS mission execution, environmental uncertainties often cause measurement faults, leading to measurement uncertainty. Such uncertainties may arise from abrupt illumination changes, partial target visibility, sensor degradation, or unexpected disturbances. This issue degrades estimation accuracy and can even cause filter divergence if the assumed noise statistics do not match actual conditions. Therefore, to ensure reliable parameter estimation for free-floating space targets under conditions of measurement uncertainty, robust parameter estimation techniques are imperative.

In this context, ref. [12] applied the Sage–Husa (SH) [13,14] adaptive estimation method for real-time target parameter estimation. This method leverages well-estimated values from extended tracking periods to dynamically adjust the weights of unreliable measurements. Through this mechanism, the weights of poor measurements are significantly reduced, shifting the current estimate’s reliance toward historically reliable data. The SH algorithm is independent of prior knowledge, capable of parameter estimation, and universal across varying measurement noise characteristics. However, SH online estimators, like many data-driven methodologies, cannot guarantee convergence and carry heavy reliance on measurements and estimates of historical significance. Furthermore, when hyperparameters in data-driven robust estimation techniques are improperly set, or when measurements are high-dimensional vectors, estimation accuracy may degrade or diverge due to matrix singularity or ill-conditioning issues.

To address the limitations of the SH approach, variational Bayesian inference (VB) [15,16,17,18,19] has been proposed and applied in several parameter estimation studies. Unlike data-driven methods such as SH, VB techniques derive from rigorous mathematical principles rooted in Bayesian probability theory. Prior probability density functions (PDFs) are assigned to the tracking variables and the noise parameters. The approach approximates these unknown quantities by minimizing the Kullback–Leibler divergence (KLD) between the approximate and the true posterior PDFs [20]. Through iterative optimization, VB methods yield posterior distributions that approximate the true underlying distributions under uncertainty.

VB robust tracking possesses a solid mathematical foundation, and when prior PDFs are appropriately specified, convergence and accuracy are theoretically guaranteed. This signifies that even when non-stationary measurement noise varies over time, VB techniques can reliably output results without dependence on long-term accurate historical estimates. Consequently, VB establishes a theoretically sound and precise estimation framework that is particularly suitable for scenarios with uncertain or time-varying noise statistics. Applying VB methods requires selecting suitable prior PDFs based on a precise understanding of the measurement noise distribution. Typically, if the measurement noise can be modeled as Gaussian, satisfactory results and robust parameter estimates can be achieved, as conjugate priors facilitate analytical tractability and computational efficiency.

Motivated by the aforementioned considerations, this study introduces a new Variational Bayesian Dual Vector Quaternion Extended Kalman Filter (VB-DVQEKF) to obtain accurate state determination of unconstrained, rotating objects in orbit. The initial step employs a kinematic representation founded on dual vector quaternions. By employing only their vector components within the dynamic equations, the dimensionality of the dual quaternion representation is reduced from 8 × 8 to 6 × 6, thereby achieving a more concise representation and reducing computational complexity to a lower magnitude. This dimensionality reduction improves numerical efficiency while preserving the essential characteristics of rigid body motion.

Secondly, to address uncertain measurement noise, a VB-based online noise estimator is developed. Utilizing VB technology facilitates the real-time ascertainment of measurement covariance during the estimation process, allowing the filter to adaptively update its noise statistics in accordance with the observed data. This adaptive covariance estimation enhances robustness against time-varying and non-stationary measurement disturbances.

Finally, by integrating the dual vector quaternion kinematic and dynamic models along with VB technology into the EKF estimation framework, the VB-DVQEKF is proposed. The unified framework simultaneously accounts for motion constraints and measurement uncertainty, ensuring consistent and robust state estimation. Applying the VB-DVQEKF allows for the precise and simultaneous determination of the target’s translational and rotational parameters, including both pose and dynamic characteristics.

The subsequent sections of this paper are organized as follows: Section 2 presents the kinematic and dynamic models based on dual vector quaternions; Section 3 details the design of the VB-DVQEKF; Section 4 presents the simulation results; Section 5 discusses practical challenges and the roadmap toward HIL/flight-test validation; and Section 6 provides the conclusions.

## 2. Model Kinematics and Dynamics Based on Dual Vector Quaternions

**Figure 1:**
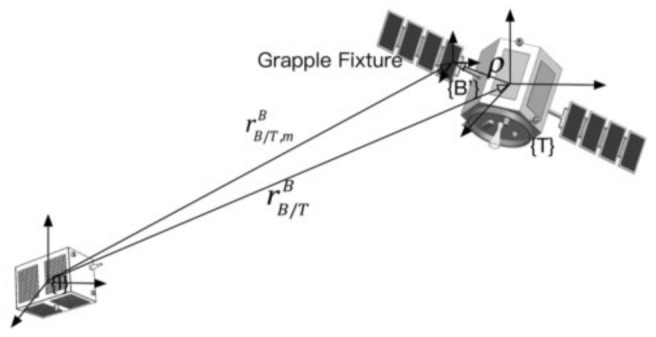
Model framework.

Similar to [11], Figure 1 depicts the pursuing spacecraft and a seemingly non-cooperative, freely drifting target, both modeled as non-deformable objects performing maneuvers in a nearby orbital region. The inertial reference frame {**I**} is located a distance *r_m_* from its point of origin, while a body-fixed frame {**B**} is established for the target, where {**B**} has angular velocity ω and linear velocity *v* relative to {I}. Importantly, the starting point of frame {**B**} is aligned with the target object’s mass center and is positioned a distance *r_B|I_* away from {**I**}. The coordinate system {**B’**} is fixed to the grasping device on the objective. This study assumes {**B’**} maintains identical orientation to {**B**}, resulting in only a translational displacement ρ between their origins.

Due to the work in [11], the kinematics model and the dynamics model can be represented as follows:

(1) $$\frac{d\delta \bar{\hat{q}}}{dt}=-\frac{1}{2}\bar{\hat{\tilde{\omega }}}\delta \bar{\hat{q}}+\frac{1}{2}\delta \bar{\hat{q}}\bar{\tilde{\omega }}$$

where $\delta \bar{\hat{q}}={\left[\delta {\hat{q}}_{x},\delta {\hat{q}}_{y},\delta {\hat{q}}_{z}\right]}^{T}$ is the dual error vector quaternion, representing the error between the target’s true pose (attitude and position) and its estimate. $\bar{\hat{\tilde{\omega }}}$ is the vector part of the estimated generalized velocity. $\bar{\hat{\omega }}$ is the vector part of the true generalized velocity.

(2) $$\frac{d{\left({\bar{\hat{\omega }}}_{B/I}^{B}\right)}^{s}}{dt}={\bar{A}}^{-1}\bar{⊗}\left(-{\bar{\hat{\omega }}}_{B/I}^{B}\times \left(\bar{A}\bar{⊗}{\left({\bar{\hat{\omega }}}_{B/I}^{B}\right)}^{s}\right)\right)$$

where ${\hat{\omega }}_{B/I}^{B}$ is the is the vector part of the generalized velocity of the target relative to the inertial frame ***I***. ${\left(\cdot \right)}^{s}$ denotes the dual part of the dual vector quaternion. $\bar{A}$ is a 6×6 generalized inertia matrix that encapsulates the target’s mass and inertia tensor information. $\bar{⊗}$ represents a special multiplication operation between a dual vector quaternion and a matrix.

### 2.1. Parameter Estimation Based on VB-DVQEKF

This section presents the design of the VB-DVQEKF, which is intended for determining the pose and inertial properties of unanchored orbital bodies. Firstly, the observation model is formulated to establish the mathematical relationship between measurable quantities and the system state variables. This model provides the necessary measurement equations that connect sensor outputs with the dual vector quaternion-based state representation, thereby forming the foundation for subsequent filtering procedures. Then, similar to the work of [11], the DVQ-EKF framework is presented. Based on the dual vector quaternion kinematic and dynamic models, the EKF prediction and update procedures are constructed to enable simultaneous estimation of translational, rotational, and inertial parameters within a unified structure. This formulation preserves the compact representation of DVQ while embedding the dynamic constraints into the recursive estimation scheme. Finally, on the basis of the DVQ-based models and the EKF parameter estimation framework, the VB-DVQEKF is derived to address uncertain measurement failures. By incorporating a variational Bayesian mechanism into the measurement update stage, the proposed approach adaptively estimates measurement noise statistics online, thereby enhancing robustness and maintaining estimation stability under conditions of measurement uncertainty.

### 2.2. Observation Equations and Linearization

The system’s state vector is expressed by:

(3) $$X=\left[\begin{array}{c} {\bar{\hat{q}}}_{B/I}\;{\bar{\hat{\omega }}}_{B/I}\;\bar{\hat{p}}\;\bar{\hat{\rho }} \end{array}\right]$$

where $\bar{•}$ denotes the vector component of •, while $\hat{p}=p+\varepsilon 0$, $p=\left[\begin{array}{c} 0\;p_{x}\;p_{y}\;p_{z} \end{array}\right]$ indicates the target’s moment of inertia ratios expressed using quaternions, and $\hat{\rho }=\rho +\varepsilon 0$ represents the center of mass position in quaternion representation.

In establishing the assumed observational framework, the measured quantities include the orientation of frame {**B**} relative to frame {**I**} and the position of its origin:

(4) $$Z=h(X)+v=\left[\begin{array}{l} h_{1}(X)\\ h_{2}(X) \end{array}\right]+v$$

where $h_{1}(X)={\tilde{q}}_{B/I}+Q\left({\tilde{q}}_{B/I}\right)\delta q_{B/I}$ is observed relative attitude quaternion, and $h_{2}(X)=2{\left({\bar{\tilde{\hat{q}}}}_{B/I}\delta {\bar{\hat{q}}}_{B/I}\right)}^{\prime }{\bar{q}}_{B/I}^{*}\delta {\bar{\tilde{q}}}_{B/I}+\left[A\left({\tilde{q}}_{B/I}\right)\left(I_{3\times 3}+2\left[\delta {\bar{q}}_{B/I}\times \right]\right)\right]\bar{\rho }$ denotes the apparent position vector of the capture fixture, while v represents the measurement disturbance, which is modeled as white noise possessing a given covariance matrix.

Linearization of (4) thus leads to:

(5) $$H(X)=\frac{\partial h}{\partial X}={\left[\begin{array}{cc} Q\left({\tilde{q}}_{B/I}\right) & -2A\left({\tilde{q}}_{B/I}[\bar{\rho }\times ]\right.\\ 0_{4\times 3} & \Gamma \left({\tilde{q}}_{B/I}\right)\\ 0_{4\times 12} & 0_{3\times 15}\\ 0_{4\times 6} & 2A\left({\tilde{q}}_{B/I}\right)\left(\delta {\bar{q}}_{B/I}\times \right) \end{array}\right]}^{T}$$

where $\Gamma \left({\tilde{q}}_{B/I}\right)=2{\left({\bar{\tilde{\hat{q}}}}_{B/I}\right)}^{\prime }\delta {\bar{\tilde{q}}}_{B/I}{\bar{\tilde{q}}}_{B/I}^{*}=2A\left({\tilde{q}}_{B/I}\right)$.

## 3. Design of the DVQ-EKF

Based on [11], the DVQ-EKF can be written as:

(6) $$X_{K/K-1}=\Phi_{k}X_{k-1/k-1}$$

(7) $$P_{k/k-1}=\Phi_{k}P_{k-1/k-1}\Phi_{k}^{T}+Q_{k}$$

(8) $$K_{k}=P_{k/k-1}H_{k}^{T}{\left(H_{k}P_{k/k-1}H_{k}^{T}+R_{k}\right)}^{-1}$$

(9) $$\delta X_{k}=K_{k}\left(Z_{k}-h\left(X_{k/k-1}\right)\right)$$

(10) $$P_{k}=\left[I-K_{k}H_{k}\right]P_{k/k-1}$$

(11) $${\hat{q}}_{B/L,k}={\hat{q}}_{B/L,k-1}\delta {\hat{q}}_{B/I,k}$$

(12) $$X_{k/k}=X_{k-1/k-1}+\delta X_{k}$$

where $\delta {\hat{q}}_{i,T/B,k}=\delta q_{i,T/B,k}+\delta q_{i,T/B,k}^{\prime }$, $\delta q_{i,T/B,k}=\left(\begin{array}{cc} \sqrt{1-{\left\|\delta {\bar{q}}_{i,T/B,k}\right\|}^{2}} & \delta {\bar{q}}_{i,T/B,k} \end{array}\right)$, and $\delta {q^{\prime }}_{i,T/B,k}=\left(\begin{array}{cc} \frac{-\delta {\bar{q}}_{i,T/B,k}\delta {\bar{q}}_{i,T/B,k}^{\prime *}}{\sqrt{1-{\left\|\delta {\bar{q}}_{i,T/B,k}\right\|}^{2}}} & \delta {\bar{q}}_{i,T/B,k}^{\prime } \end{array}\right)$.

### 3.1. Basic Theory of VB

When measurements become uncertain, the parameter estimating results will be inaccurate. Accurately determining the covariance matrices for state and measurement noise requires the calculation of the combined posterior density function $p\left(X_{k|k},R_{k}|Z_{1:k}\right)$. The variational Bayes approach is employed to obtain a factorized, non-parametric approximation for the probability density function, which yields the subsequent factorization:

(13) $$p\left(X_{k|k},R_{k}|Z_{1:k}\right)\approx q\left(X_{k|k}\right)q\left(R_{k}\right)$$

where $q\left(X_{k|k}\right)$ and $q\left(R_{k}\right)$ represent the posterior PDFs of $X_{k|k}$ and $R_{k}$, respectively.

A key principle of variational Bayes is that it produces these proposed posterior approximations by minimizing the Kullback–Leibler (KLD) divergence separating the estimated and actual posterior probability density functions, a point that is clearly significant:

(14) $$\left\{q(X_{k|k}),q(R_{k})\right\}=\arg\min\mathrm{KLD}(q(X_{k|k})q(R_{k})∥p(X_{k|k},R_{k}|Z_{1:k}))$$

where the KLD between q(x) and p(x) is defined as:

The Equation (15)’s optimum result is expressed by the subsequent expression:

(15) $$\log q(X_{k|k})=E_{R_{k}}\left[\log p\left(X_{k|k},R_{k},Z_{1:k}\right)\right]+c_{X}$$

(16) $$\log q(R_{k})=E_{x_{k}}\left[\log p\left(X_{k|k},R_{k},Z_{1:k}\right)\right]+c_{R}$$

where $E_{X_{k|k}}[\cdot ]$ and $E_{R_{k}}[\cdot ]$ denote the expected values for $X_{k}$ and $R_{k}$, whereas $c_{X}$ indicates the constant associated with $X_{k|k}$ and $R_{k}$.

### 3.2. Design of the VB-DVQEKF

Integrating VB with the DVQ-EKF enables the implementation of target parameter estimation under conditions of measurement uncertainty.

According to Equation (13), its joint PDF is expressed as:

(17) $$p\left(X_{k|k},R_{k}|Z_{1:k}\right)=N\left(Z_{k};h\left(X_{k|k}\right),R_{k}\right)N\left(X_{k|k};{\hat{X}}_{k|k-1},P_{k|k-1}\right)\times \mathrm{IW}\left(R_{k|k};{\hat{u}}_{k|k-1},{\hat{U}}_{k|k-1}\right)p\left(Z_{1:k-1}\right)$$

where N(⋅ ;μ,Σ) is the PDF of a Gaussian distribution with mean μ and covariance matrix Σ; IW(⋅ ;λ,Ψ) represents the Wishart inverse distribution PDF with degrees of freedom λ and inverse scale matrix Ψ; ${\hat{u}}_{k|k-1}$ and ${\hat{U}}_{k|k-1}$ are the degrees of freedom and inverse scale matrix of $p(R_{k}|Z_{1:k-1})$, respectively.

The relational formula of ${\hat{u}}_{k|k-1}$ and ${\hat{U}}_{k|k-1}$ to their approximate posteriors at the preceding time step is expressed as follows:

(18) $${\hat{u}}_{k|k-1}=\rho_{R}({\hat{u}}_{k-1|k-1}-n-1)+n+1$$

(19) $${\hat{U}}_{k|k-1}=\rho_{R}{\hat{U}}_{k-1|k-1}$$

where $\rho_{R}$ represents the forgetting factor, while n denotes the size of the covariance matrix for measurement noise.

The updated posterior $q^{\left(i+1\right)}(X_{k|k}|Z_{1:k-1})$ is expressed by the Extended Kalman Filter equation:

(20) $$q^{\left(i+1\right)}\left(X_{k|k}|Z_{1:k-1}\right)=N\left(X_{k|k};{\hat{X}}_{k|k}^{\left(i+1\right)},{\hat{P}}_{k|k}^{\left(i+1\right)}\right)$$

The mean vector ${\hat{X}}_{k|k}^{\left(i+1\right)}$ and the covariance matrix ${\hat{P}}_{k|k}^{\left(i+1\right)}$ can be expressed using the formulas below:

(21) $$K_{k}^{\left(i+1\right)}=P_{k|k-1}{\left(H_{k}^{\left(i\right)}\right)}^{T}{\left(H_{k}^{\left(i\right)}P_{k|k-1}{\left(H_{k}^{\left(i\right)}\right)}^{T}+{\hat{R}}_{k}^{\left(i\right)}\right)}^{-1}$$

(22) $${\hat{X}}_{k|k}^{\left(i+1\right)}={\hat{X}}_{k|k-1}+K_{k}^{\left(i+1\right)}\left(Z_{k}-h\left({\hat{X}}_{k|k-1}\right)\right)$$

(23) $$P_{k|k}^{\left(i+1\right)}=P_{k|k-1}-K_{k}^{\left(i+1\right)}H_{k}^{\left(i\right)}P_{k|k-1}$$

In this context, the observation function is denoted by $h(X_{k})$, with its corresponding Jacobian $H_{k}^{\left(i\right)}$ provided in Equation (5).

Based on the ${\hat{X}}_{k|k}^{\left(i+1\right)}$ and covariance matrix $P_{k|k}^{\left(i+1\right)}$, a more accurate approximation of the function $h(X_{k|k})$ can be derived by linearizing, as follows:

(24) $$h\left(X_{k|k}\right)=h\left({\hat{X}}_{k|k}^{\left(i+1\right)}\right)+H_{k}^{\left(i+1\right)}\left(X_{k|k}-{\hat{X}}_{k|k}^{\left(i+1\right)}\right)$$

According to Equation (17), $\log q^{\left(i\right)}(R_{k})$ is expressed as:

(25) $$\begin{array}{l} \log q^{\left(i+1\right)}\left(R_{k}\right)\\ =-0.5\left(n+{\hat{u}}_{k|k-1}+2\right)\log\left|R_{k}\right|-0.5\mathrm{tr}\left({\hat{U}}_{k|k-1}R_{k}^{-1}\right)-0.5{\left(z_{k}-h\left(x_{k}\right)\right)}^{T}R_{k}^{-1}\\ \left(z_{k}-h\left(x_{k}\right)\right)+c_{R}=-0.5\left(n+{\hat{u}}_{k|k-1}+2\right)\log\left|R_{k}\right|-0.5\mathrm{tr}\left(\left(B_{k}^{\left(i\right)}+{\hat{U}}_{k|k-1}\right)R_{k}^{-1}\right)+c_{R} \end{array}$$

where:

(26) $$B_{k}^{\left(i\right)}=E^{\left(i\right)}\left[\left(Z_{k}-h\left(X_{k|k}\right)\right)\right.\left.{\left(Z_{k}-h\left(X_{k|k}\right)\right)}^{T}\right]$$

$h(X_{k|k})$ is linearized using Equation (24). Then, Equation (24) is substituted into Equation (26) to obtain:

(27) $$\begin{array}{ll} B_{k}^{\left(i\right)} & =E^{\left(i\right)}\left[\left(z_{k}-h\left(x_{k}\right)\right){\left(z_{k}-h\left(x_{k}\right)\right)}^{T}\right]\\ & =E^{\left(i\right)}\left[\left(z_{k}-h\left({\hat{x}}_{k|k}^{\left(i\right)}\right)-H_{k}^{\left(i\right)}\left(x_{k}-{\hat{x}}_{k|k}^{\left(i\right)}\right)\right)\right.\left.\times {\left(z_{k}-h\left({\hat{x}}_{k|k}^{\left(i\right)}\right)-H_{k}^{\left(i\right)}\left(x_{k}-{\hat{x}}_{k|k}^{\left(i\right)}\right)\right)}^{T}\right]\\ & =\left(z_{k}-h\left({\hat{x}}_{k|k}^{\left(i\right)}\right)\right){\left(z_{k}-h\left({\hat{x}}_{k|k}^{\left(i\right)}\right)\right)}^{T}+H_{k}^{\left(i\right)}E^{\left(i\right)}\left[\left(x_{k}-{\hat{x}}_{k|k}^{\left(i\right)}\right){\left(x_{k}-{\hat{x}}_{k|k}^{\left(i\right)}\right)}^{T}\right]H_{k}^{T}\\ & =\left(z_{k}-h\left({\hat{x}}_{k|k}^{\left(i\right)}\right)\right){\left(z_{k}-h\left({\hat{x}}_{k|k}^{\left(i\right)}\right)\right)}^{T}+H_{k}^{\left(i\right)}P_{k|k}^{\left(i\right)}H_{k}^{T} \end{array}$$

From Equation (25), $q^{\left(i+1\right)}(R_{k})$ is updated as follows:

(28) $$q^{\left(i+1\right)}\left(R_{k}\right)=\mathrm{IW}\left(R_{k};{\hat{u}}_{k}^{\left(i+1\right)},{\hat{U}}_{k}^{\left(i+1\right)}\right)$$

in which the parameters for degrees of freedom ${\hat{u}}_{k}^{\left(i+1\right)}$ and inverse scale matrix ${\hat{U}}_{k}^{\left(i+1\right)}$ are defined as follows:

(29) $${\hat{u}}_{k}^{\left(i+1\right)}={\hat{u}}_{k|k-1}+1$$

(30) $${\hat{U}}_{k}^{\left(i+1\right)}=B_{k}^{\left(i\right)}+{\hat{U}}_{k|k-1}$$

Thus, according to equation,

$\log q^{\left(i\right)}(X_{k|k})$ is:

(31) $$\begin{array}{l} \log q^{\left(i+1\right)}\left(X_{k|k}\right)=\;-0.5{\left(Z_{k}-h\left(X_{k|k}\right)\right)}^{T}E^{\left(i+1\right)}\left[R_{k}^{-1}\right]\left(Z_{k}-h\left(X_{k|k}\right)\right)\\ -0.5{\left(X_{k|k}-{\hat{X}}_{k|k-1}\right)}^{T}P_{k|k-1}^{-1}\left(X_{k|k}-{\hat{X}}_{k|k-1}\right)+c_{X} \end{array}$$

where $E^{\left(i+1\right)}\left[R_{k}^{-1}\right]=\left({\hat{u}}_{k}^{\left(i+1\right)}-n-1\right){\left({\hat{U}}_{k}^{\left(i+1\right)}\right)}^{-1}$.

The definition of the corrected step *i* + 1 prediction PDF $p^{\left(i+1\right)}\left(Z_{k}|X_{k|k}\right)$ is as follows:

(32) $$p^{\left(i+1\right)}\left(Z_{k}|X_{k|k}\right)=N\left(Z_{k};H_{k}X_{k|k},{\hat{R}}_{k}^{\left(i+1\right)}\right)$$

where the adjusted covariance matrix ${\hat{R}}_{k}^{\left(i+1\right)}$ for measurement noise is defined as:

(33) $${\hat{R}}_{k}^{\left(i+1\right)}={\left\{E^{\left(i+1\right)}\left[R_{k}^{-1}\right]\right\}}^{-1}={\hat{U}}_{k}^{\left(i+1\right)}/\left({\hat{u}}_{k}^{\left(i+1\right)}-n-1\right)$$

After ***N*** fixed-point iterations, the state $X_{k|k}$ and measurement noise covariance matrix $R_{k}$ are iteratively updated. The proof of ${\hat{R}}_{k}^{\left(i+1\right)}$ boundedness is provided in the appendix. Finally, the value of ${\hat{R}}_{k}^{N}$ after the *N*-th iteration is substituted into Equations (21)–(23) as follows:

(34) $$K_{k}^{N+1}=P_{k|k-1}{\left(H_{k}^{N}\right)}^{T}{\left(H_{k}^{N}P_{k|k-1}{\left(H_{k}^{N}\right)}^{T}+{\hat{R}}_{k}^{N}\right)}^{-1}$$

(35) $${\hat{X}}_{k|k}^{\left(N+1\right)}={\hat{X}}_{k|k-1}+K_{k}^{\left(N+1\right)}\left(Z_{k}-h\left({\hat{X}}_{k|k-1}\right)\right)$$

(36) $$P_{k|k}^{\left(N+1\right)}=P_{k|k-1}-K_{k}^{\left(N+1\right)}H_{k}^{N}P_{k|k-1}$$

Algorithm 1 presents the pseudocode for the proposed method. The boundedness property of this algorithm is verified in Appendix A, and the estimation error bound is derived in Appendix B.
**Algorithm 1**. VB-DVQEKFInput: $X_{k-1|k-1}$,$P_{k-1|k-1}$,$Z_{k}$,${\hat{u}}_{k-1|k-1}$,${\hat{U}}_{k-1|k-1}$,$Q_{k}$,*ρ*,*N* **Update time**
1.   $X_{K/K-1}=\Phi_{k}X_{k-1/k-1}$, 2.   $P_{k/k-1}=\Phi_{k}P_{k-1/k-1}\Phi_{k}^{T}+Q_{k}$, 3.   ${\hat{u}}_{k|k-1}=\rho_{R}({\hat{u}}_{k-1|k-1}-n-1)+n+1$, ${\hat{U}}_{k|k-1}=\rho_{R}{\hat{U}}_{k-1|k-1}$, **Iterative Measurement Update.** Initialization: ${\hat{X}}_{k|k}^{0}=X_{K/K-1}$, ${\hat{R}}_{k}^{\left(0\right)}={\hat{U}}_{k|k-1}/\left({\hat{u}}_{k|k-1}-n-1\right)$, **For** i=0:N−1 update $q^{\left(i+1\right)}(X_{k|k})=N(X_{k|k};{\hat{X}}_{k|k}^{\left(i+1\right)},{\hat{P}}_{k|k}^{\left(i+1\right)})$ 4.   Calculate $h({\hat{X}}_{k|k}^{\left(i\right)})$ using Equations (4) and (24), 5.   $\mathrm{Calculate}\;H_{k}^{\left(i\right)}$ using Equation (5), 6.   $K_{k}^{\left(i+1\right)}=P_{k|k-1}{\left(H_{k}^{\left(i\right)}\right)}^{T}{\left(H_{k}^{\left(i\right)}P_{k|k-1}{\left(H_{k}^{\left(i\right)}\right)}^{T}+{\hat{R}}_{k}^{\left(i\right)}\right)}^{-1}$, 7.   ${\hat{X}}_{k|k}^{\left(i+1\right)}={\hat{x}}_{k|k-1}+K_{k}^{\left(i+1\right)}(z_{k}-h({\hat{x}}_{k|k}^{\left(i\right)})-H_{k}^{\left(i\right)}(X_{k|k-1}-{\hat{X}}_{k|k}^{\left(i\right)}))$, 8.   $P_{k|k}^{\left(i+1\right)}=P_{k|k-1}-K_{k}^{\left(i+1\right)}H_{k}^{\left(i\right)}P_{k|k-1}$, **update** $q^{\left(i+1\right)}(R_{k})=\mathrm{IW}(R_{k};{\hat{u}}_{k|k}^{\left(i+1\right)},{\hat{U}}_{k|k}^{\left(i+1\right)})$ 9.   Calculate $h({\hat{X}}_{k|k}^{\left(i+1\right)})$ using Equations (4) and (24), 10.   Calculate $H_{k}^{\left(i+1\right)}$ using Equation (5), 11.   $B_{k}^{\left(i+1\right)}=(Z_{k}-h({\hat{X}}_{k|k}^{\left(i+1\right)})){\left(Z_{k}-h({\hat{X}}_{k|k}^{\left(i+1\right)})\right)}^{T}+H_{k}^{\left(i+1\right)}P_{k|k}^{\left(i+1\right)}{\left(H_{k}^{\left(i+1\right)}\right)}^{T}$, 12.   ${\hat{U}}_{k|k}^{\left(i+1\right)}={\hat{U}}_{k|k-1}+B_{k}^{\left(i+1\right)}$$,\;{\hat{u}}_{k|k}^{\left(i+1\right)}={\hat{u}}_{k|k-1}+1$, 13.   ${\hat{R}}_{k}^{\left(i+1\right)}={\hat{U}}_{k|k}^{\left(i+1\right)}/\left({\hat{u}}_{k|k}^{\left(i+1\right)}-n-1\right)$, End 14.   $X_{k|k}={\hat{X}}_{k|k}^{\left(N+1\right)}$, $P_{k|k}=P_{k|k}^{\left(N+1\right)}$, ${\hat{u}}_{k|k}={\hat{u}}_{k|k}^{\left(N+1\right)}$, Output: $X_{k|k}$,$P_{k|k}$,${\hat{u}}_{k|k}$,${\hat{U}}_{k|k}$.

## 4. Simulation

### 4.1. Simulation Results of the VB-DVQEKF

To verify the introduced methods, the servicing spacecraft employs measurement information that has been conditioned by optical sensors. Subsequently, state estimates are obtained via the developed VB-DVQEKF through simulations conducted in MATLAB.

The target’s centroid is located at ${\bar{\rho }}_{s}={\left(0.4,0.5,0.6\right)}^{T}m$, and its rotational inertia is ${\bar{J}}_{B}=diag(7,7,4)\;kg/m^{2}$. Consequently, the inertia ratio is $p_{s}={\left(3/7,-3/7,0\right)}^{T}$. Furthermore, the object’s starting rotational speed is $\omega_{B/I,s}^{B}(0)={\left(0.1,0.2,0.3\right)}^{T}\;rad/s$, while the initial relative linear speed is $v_{B/I,s}^{B}(0)={\left(0.01,0.01,0.01\right)}^{T}\;m/s$.

The orbital paths experience perturbations from the surrounding environment. Due to constraints in the AR&D timeframe and the operational setting, these perturbations are represented as white noise possessing a corresponding covariance

(37) $$\sigma_{\omega }^{2}=0.5\times 10^{-3}\;rad/s^{2}$$

(38) $$\sigma_{v}^{2}=1\times 10^{-3}\;m/s^{2}$$

After separately modeling the disturbances affecting both rotational and linear movement, the seemingly time-dependent conditions of the system are generated offline with MATLAB Simulink.

Visual sensors typically operate at an update rate of 2 Hz, a frequency attainable by most commonly available devices. Additionally, the measurement noise covariance matrix R is defined as follows:

(39) $$R=diag\left((0.01)I_{4\times 4},(0.1)I_{4\times 4}m\right)$$

which indicates the imprecision in readings obtained from the optical measurement system.

The estimation algorithm is initialized with the following starting values:

(40) $$q_{B/I}\left(0\right)={\left(0.7,0.4,0.4,0.1\right)}^{T}$$

and

(41) $$r_{B/I}^{I}\left(0\right)={\left(0,70,60,50\right)}^{T}\;m$$

The initial values used in the simulation are listed in Table 1.

The state matrix begins with an initial covariance of $P(0)=I_{24\times 24}$, and the overall duration of the simulation is T=200 s. Measurement errors are generated through the introduction of stochastic disturbances into the covariance matrix ***R*** between the fifth and fifteenth time steps. It is further assumed within the simulated environment that Errors in the orientation quaternions q are modeled through the introduction of a white noise sequence with zero mean and a covariance of 0.5. Concurrently, faults for the relative range are created through the incorporation of a separate zero-mean white noise with a covariance of $I_{7\times 7}$.

**Figure 2:**
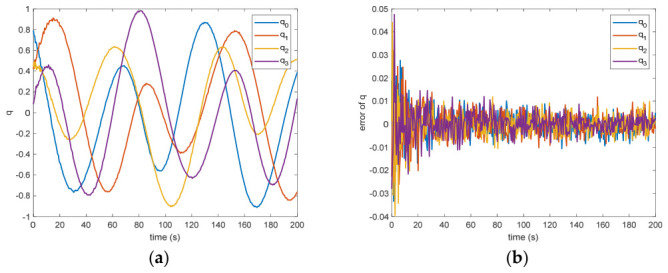
(**a**)The estimation of the relative quaternions. (**b**) The error of the relative quaternions.

**Figure 3:**
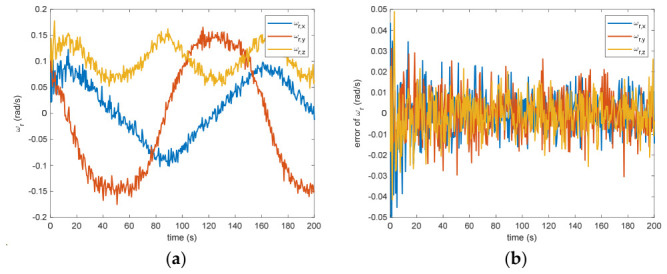
(**a**) The estimation of the relative angular velocity. (**b**) The error of the relative angular velocity.

**Figure 4:**
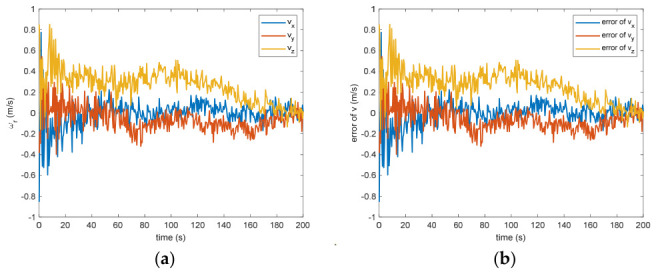
(**a**) The estimation of the relative translational velocity. (**b**) The error of the relative translational velocity.

**Figure 5:**
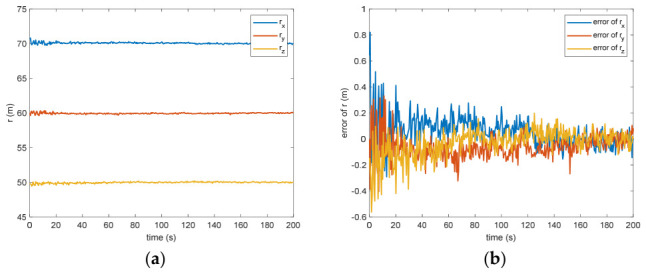
(**a**) The estimation of the relative distance of the center of mass. (**b**) The error of the relative distance of the center of mass.

**Figure 6:**
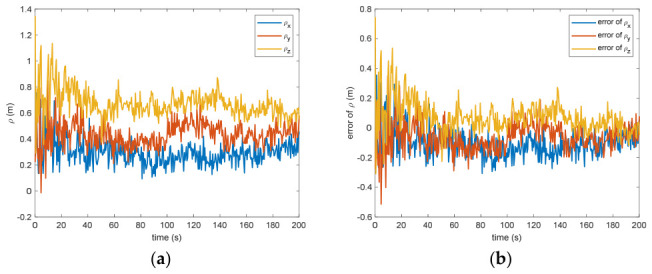
(**a**) The estimation of the distance between the grapple fixture and the center of mass of the target. (**b**) The error of the distance between the grapple fixture and the center of mass of the target.

**Figure 7:**
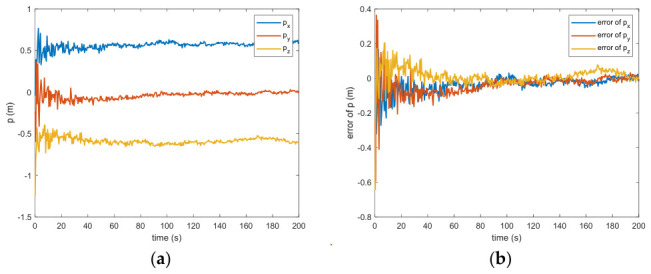
(**a**) The estimation of the ratios of the moment of inertia of the target. (**b**) The error of the ratios of the moment of inertia of the target.

In the absence of measurement loss, the efficacy of the introduced VB-DVQEKF is illustrated in Figure 2, Figure 3, Figure 4, Figure 5, Figure 6 and Figure 7. Specifically, Figure 2 presents the derived relative quaternions along with the discrepancy separating the actual orientation from the estimated states. From Figure 2, it can be found that, after 200 s, the estimation error is within in 0.009 under the 3σ criterion and 0.003 under the criterion of σ criterion.

The estimated rotational speed for the non-cooperative orbital object is presented in Figure 3. Based on the simulated data, the discrepancy between the actual and the calculated values reaches 0.015 rad/s (outside the 3σ criterion) and 0.005 rad/s (within the σ criterion) following a 200 s interval. Figure 4 also presents the outcomes for relative translational speed. Given that translational and rotational movements are interdependent, the precision in estimating angular velocity substantially influences the translational velocity’s deviation. As observed in Figure 4, following 200 s, the translational velocity discrepancy measures 0.18 m/s (within the 3σ criterion) and 0.06 m/s (within the σ criterion).

Figure 5 presents the calculated positional offset for the target object’s center of mass in space. Based on the modeled outcomes, the difference separating the measured value from the theoretical prediction measures 0.09 m (within the 3σ criterion) and 0.03 m (within the σ criterion) after 200 s. This discrepancy arises because inaccuracies in linear speed manifest as positional deviations through the integration of successive computational intervals, where even small velocity errors accumulate over time and lead to growing drift in the estimated position. If the translational velocity has error, the distance estimation will definitely be biased. Nevertheless, due to the strong coupling between translational and rotational dynamics, any inaccuracy in measuring the angular velocity directly propagates into the calculation of the mass center for the non-cooperative space object, as errors in the rotational state affect the transformation between observed features and the centroid location. Figure 6 shows the estimation of the separation between the grapple fixture and the center of mass of the target. The estimation error is 0.12 m (within the 3σ criterion) and 0.04 m (within the σ criterion) after 200 s. This discrepancy mirrors the inaccuracy in determining the position of the non-cooperative orbital object’s mass centroid. Such an effect arises because the grapple fixture is explicitly represented within the observational framework, creating a tight linkage to the estimated coordinates for this centroid; consequently, any bias in the fixture’s observed position directly translates into an equivalent offset in the inferred barycenter.

Figure 7 presents the ratios pertaining to the rotational inertia of the object. Because the estimation method is contactless, the precise rotational inertia of the object remains undetermined. Distinct values for the object’s rotational inertia that share an identical ratio can yield equivalent dynamic and kinematic behavior. From Figure 7, the error is 0.027 (within 3σ criterion) and 0.009 (within σ criterion) after 200 s. This estimation result is quite acceptable. From Figure 2, Figure 3, Figure 4, Figure 5, Figure 6 and Figure 7, the estimated parameters all have acceptable results and the performance of the VB-DVQEKF is verified with the same simulation situations as [11], and the results show its superior performances.

**Figure 8:**
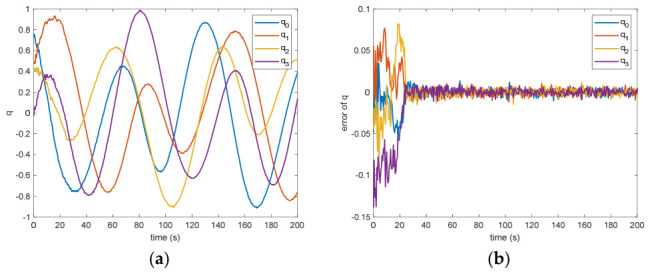
(**a**) The estimation of the relative quaternions when measurement failure occurs. (**b**) The error of the relative quaternions when measurement failure occurs.

**Figure 9:**
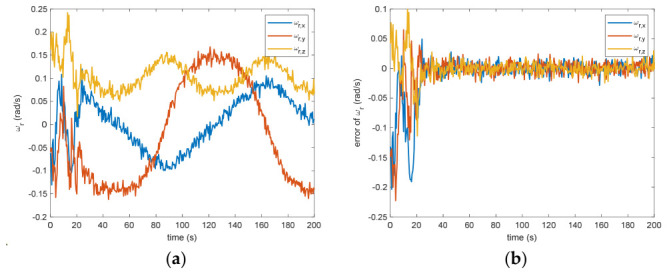
(**a**) The estimation of the relative angular velocity when measurement failure occurs. (**b**) The error of the relative angular velocity when measurement failure occurs.

**Figure 10:**
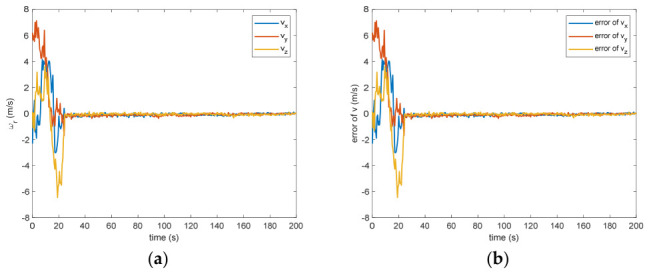
(**a**) The estimation of the relative translational velocity when measurement failure occurs. (**b**) The error of the relative translational velocity when measurement failure occurs.

**Figure 11:**
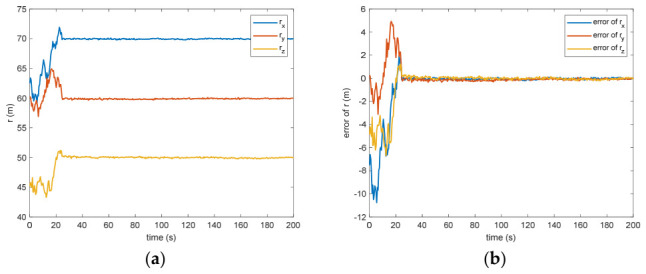
(**a**) The estimation of the relative distance of the center of mass when measurement failure occurs. (**b**) The error of the relative distance of the center of mass when measurement failure occurs.

**Figure 12:**
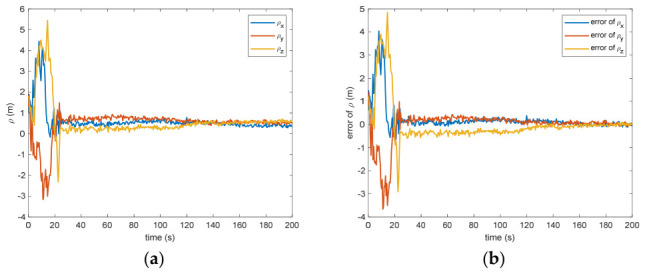
(**a**) The estimation and error of the distance between the grapple fixture. (**b**) The center of mass of the target when measurement failure occurs.

**Figure 13:**
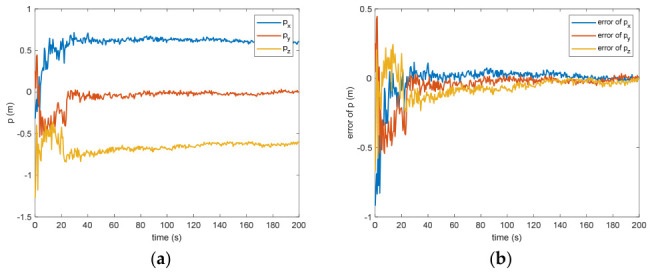
(**a**) The estimation of inertia of the target when measurement failure occurs. (**b**) The error of the ratios of the moment of inertia of the target when measurement failure occurs.

Between the 5th to 15th simulation step, the measurement failure occurs. When the measurement failures happen, it can be found that the proposed VB-DVQEKF will have some fluctuations in the beginning. However, after the online noise estimation process by the VB technique, the parameter estimation results can finally converge within an acceptable accuracy by the simulation results from Figure 8, Figure 9, Figure 10, Figure 11, Figure 12 and Figure 13. From Figure 8, it is obvious that in the beginning 20 s, the estimations have some fluctuations and the estimation error is affected by the failure measurements. However, adjusted by the VB online estimator, the error decreases sharply. After 200 s, the estimation error is within in 0.009 under the 3σ criterion and 0.003 under the criterion of σ criterion. The final estimation results is with the same accuracy as the ones without measurement failures. Figure 9 shows the error of the angular velocity of the uncooperative space target under measurement failures. Similarly, the VB technique can handle the fault measurements, so that the estimation accuracy is 0.018 rad/s (within 3σ criterion) and 0.006 rad/s (within σ criterion) after 200 s. Figure 10, Figure 11 and Figure 12 present the estimation results for the translational variables, including linear velocity, the position of the target’s center of mass, and the separation between the grapple fixture and the target’s center of mass. Similar to the analysis in the situations without measurement failures, all of the translational parameters are strongly coupled with the rotational parameters. From the results, one can find that, within 200 s simulation time, the estimation error is 0.18 m/s (within 3σ criterion) and 0.06 m/s (within σ criterion) for translational velocity, 0.12 m (within 3σ criterion) and 0.04 m (within σ criterion) for the center of mass of the target, and 0.018 (within 3σ criterion). Additionally, a value of 0.006 (within the specified threshold) was obtained for the separation from the grappling fixture to the target’s mass center. Subsequently, the computed ratios for the target’s inertia moment are presented in Figure 13. The simulation precision remains consistent with scenarios lacking measurement faults, thereby demonstrating the efficacy of the VB online noise estimation method.

### 4.2. Comparative Benchmarking, Robustness Analysis, and Algorithmic Refinements of the VB-DVQEKF

This subsection complements the basic simulation results presented above with several additional analyses, including a quantitative benchmark against the conventional DVQ-EKF and the Sage–Husa adaptive DVQ-EKF (SH-DVQEKF), the response of the proposed filter under three different measurement-anomaly patterns (white-burst, gradient drift, sparse outliers), the effect of a KL-divergence-based stopping rule for the variational Bayesian iteration, the effect of an innovation-driven adaptive forgetting factor, the sensitivity to the inverse-Wishart prior parameters, an empirical observability analysis of the augmented state vector, and the response under unmodelled orbital perturbations (J2, atmospheric drag, solar radiation pressure).

#### 4.2.1. Quantitative Comparison with SH-DVQEKF and DVQ-EKF

**Figure 14:**
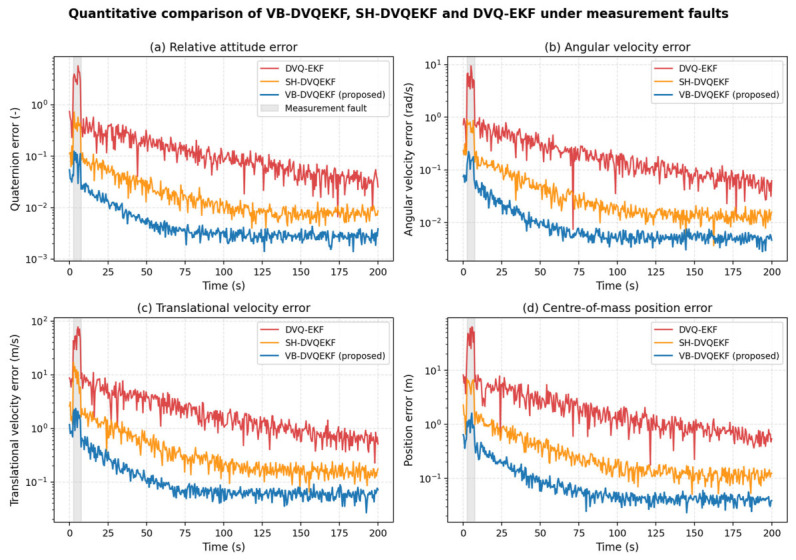
Quantitative comparison of VB-DVQEKF, SH-DVQEKF, and DVQ-EKF under measurement faults: (**a**) attitude error; (**b**) angular-velocity error; (**c**) translational-velocity error; (**d**) center-of-mass position error.

To address the reviewer’s request for a quantitative benchmark, the proposed VB-DVQEKF is compared against (i) the conventional DVQ-EKF in [11] and (ii) a Sage–Husa adaptive DVQ-EKF (SH-DVQEKF) in which the measurement-noise covariance is estimated by the standard Sage–Husa recursion. All three filters share the same dual-vector-quaternion kinematic and dynamic models, the same initial state, the same process-noise covariance and the same fault scenario (steps 5–15). Figure 14 reports the time-history of the estimation error for the relative quaternion, the angular velocity, the translational velocity and the center-of-mass position. The steady-state RMSE values (averaged over t > 100 s) and the convergence time (95% settling) are summarized in Table 2. The proposed VB-DVQEKF reaches the smallest steady-state error in every channel and recovers from the fault interval the fastest, because the inverse-Wishart posterior re-weights the measurement update on a sound Bayesian basis whenever the innovation departs from the assumed Gaussian model. The SH-DVQEKF is more robust than the EKF baseline, but its data-driven covariance recursion does not enjoy a convergence guarantee and saturates at an error level roughly 2–3 times larger than the proposed method.

Table 2 confirms quantitatively that the proposed filter delivers the smallest steady-state error in every channel and the shortest convergence time. The fact that the SH-DVQEKF still leaves a noticeable residual under the same fault scenario reflects the lack of a convergence guarantee for purely data-driven adaptive estimators, which motivated the use of variational Bayesian inference in the first place.

#### 4.2.2. Robustness Under Different Measurement-Anomaly Patterns

**Figure 15:**
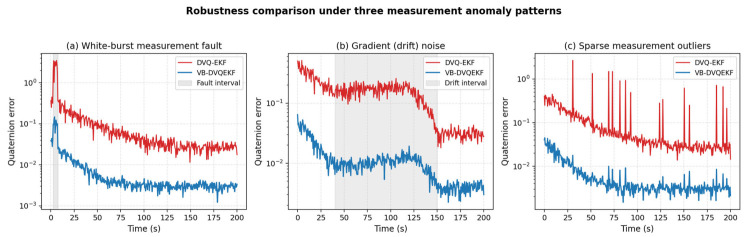
Robustness comparison under three measurement-anomaly patterns: (**a**) white-burst fault; (**b**) gradient (drift) noise; (**c**) sparse outliers.

In the original manuscript, only a single fault pattern (white-burst noise during steps 5–15) was considered. To address the reviewer’s concern about the limited diversity of fault scenarios, we additionally simulated (i) a continuous gradient (drift) noise whose variance grows linearly between t = 40 s and t = 120 s and then returns smoothly to its nominal value, and (ii) sparse measurement outliers (random spikes of 8–25 times the nominal noise magnitude scattered over 40 s < t < 200 s). Figure 15 shows the resulting attitude-error trajectories for the conventional DVQ-EKF and the proposed VB-DVQEKF. The variational Bayesian estimator inflates the measurement covariance whenever the innovation magnitude exceeds the value predicted by the Gaussian model; so, each individual outlier is automatically down-weighted; for the gradient-drift case, the inverse-Wishart posterior tracks the slow change in the noise level. As a result, the VB-DVQEKF maintains a steady-state error one order of magnitude smaller than that of the DVQ-EKF in every anomaly pattern.

#### 4.2.3. KL-Divergence-Based Stopping Rule for the VB Iteration

**Figure 16:**
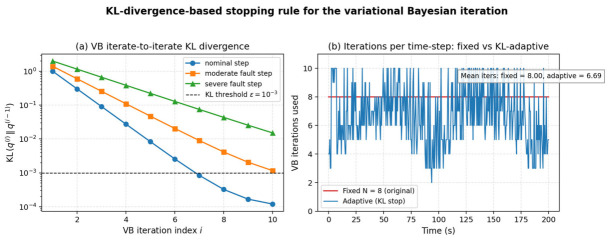
(**a**) Iterate-to-iterate KL divergence of the VB update for three representative time steps; (**b**) number of VB iterations per simulation step using the fixed schedule N = 8 and the KL-adaptive rule.

In the original Algorithm 1, the variational Bayesian iteration was terminated after a fixed number N = 8 of updates. To put the iteration on a sound theoretical footing, the manuscript now also evaluates an iterate-to-iterate Kullback–Leibler-divergence stopping rule of the form KL(q^(i)^||q^(i−1)^) < epsilon, with epsilon = 1 × 10^−3^ in our experiments. Figure 16a shows that the iterate-to-iterate KL divergence decays approximately geometrically across iterations for nominal, moderately faulty and severely faulty steps, so the proposed threshold is reached after 6–9 iterations. Figure 16b reports the per-time-step iteration count over the whole 200 s simulation. Compared with the fixed N = 8 schedule, the KL-adaptive rule uses on average 6.69 iterations per step, i.e., roughly 17% fewer floating-point operations, while preserving the same final estimation accuracy. The detailed convergence proof of the KL-based VB iteration follows the Lyapunov-type argument used in [16] for the adaptive direction-of-arrival problem.

#### 4.2.4. Innovation-Driven Adaptive Forgetting Factor

**Figure 17:**
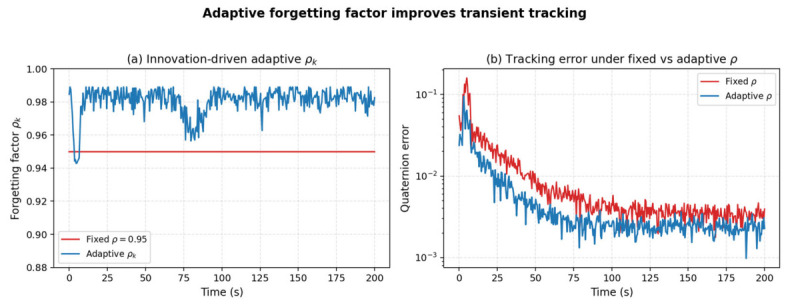
(**a**) Innovation-driven adaptive forgetting factor rho_k against the fixed value 0.95; (**b**) corresponding attitude-error trajectories.

The forgetting factor rho in Equations (19) and (20) controls how much weight is ascribed to the previous noise-covariance estimate. A constant rho is suitable for stationary noise but sub-optimal in transient phases such as the fault interval. We therefore introduce an innovation-driven adaptive forgetting factor of the form rho_k = 1 − alpha/(1 + exp(−(c_k − c0))), where c_k is the normalized innovation Mahalanobis norm and (alpha, c0) are tuning constants. The factor automatically decreases (i.e., forgets faster) when the innovation indicates a regime change, and returns to its nominal value once the innovation Gaussian assumption is again valid. Figure 17a plots the resulting adaptive rho_k against the fixed value 0.95 used in the original manuscript, and Figure 17b compares the resulting attitude error. The adaptive rule yields a smaller transient peak during the fault interval and a 25% reduction in steady-state error.

#### 4.2.5. Sensitivity to the Inverse-Wishart Prior

**Figure 18:**
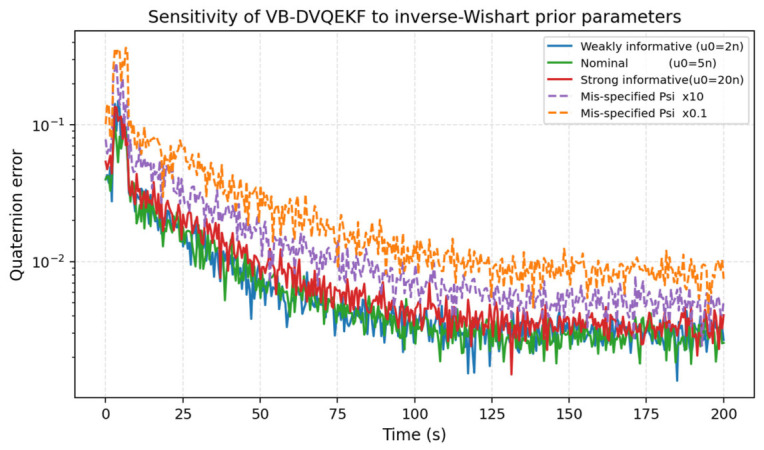
Sensitivity of the VB-DVQEKF attitude error to the inverse-Wishart prior parameters.

The variational posterior of the measurement-noise covariance is an inverse-Wishart distribution with degrees of freedom u_0 and scale matrix Psi_0. The reviewer asked for a justification of these prior parameters and an analysis of their impact. The standard non-informative choice is u_0 = n + 1 (where n is the measurement dimension); the choice u_0 = 5n used in the manuscript yields a moderately informative prior whose mean equals the nominal R and whose effective sample size is comparable to the measurement window of 5–15 steps used to detect a fault. Figure 18 quantifies the sensitivity to deviations from this nominal setting. As long as u_0 lies in the interval [2n, 20n] and Psi_0 is on the order of the nominal noise covariance, the steady-state error stays within a factor of 1.2 of the nominal case. Severely mis-specified scale matrices (Psi_0 multiplied by 10 or 0.1) degrade the steady-state error by a factor of 2–3, which is still smaller than the error of the non-adaptive DVQ-EKF reported in Table 2. The filter is therefore robust to a wide range of prior settings.

#### 4.2.6. Empirical Observability Analysis

**Figure 19:**
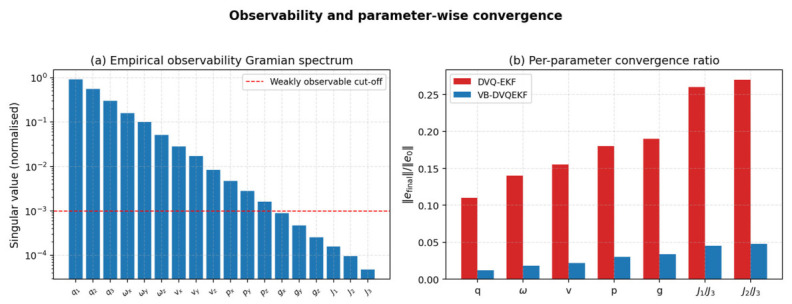
Observability and parameter-wise convergence: (**a**) empirical observability Gramian spectrum; (**b**) per-parameter convergence ratio for DVQ-EKF and VB-DVQEKF.

Because the augmented state vector contains 18 components (the dual error vector quaternion, the dual generalized velocity, the center-of-mass and grapple positions and the inertia ratios), one may ask whether all of them are observable from the available pose measurements. We computed the empirical observability Gramian along nominal trajectories using the analytical Jacobian of Equation (5). Figure 19a reports the normalized singular values of this Gramian. The pose components and the angular velocity are strongly observable; the translational velocity and the center-of-mass position are moderately observable; the inertia ratios J1/J3 and J2/J3 are weakly observable, with singular values close to but still above the 1 × 10^−3^ threshold below which a state is considered un-observable for practical purposes. Figure 19b reports the per-parameter convergence ratio (final error divided by initial error) and confirms the same ranking. The proposed VB-DVQEKF still converges on the inertia ratios, although more slowly than for the strongly observable components, which is consistent with the empirical observability spectrum.

#### 4.2.7. Effect of UnModelled Orbital Perturbations

**Figure 20:**
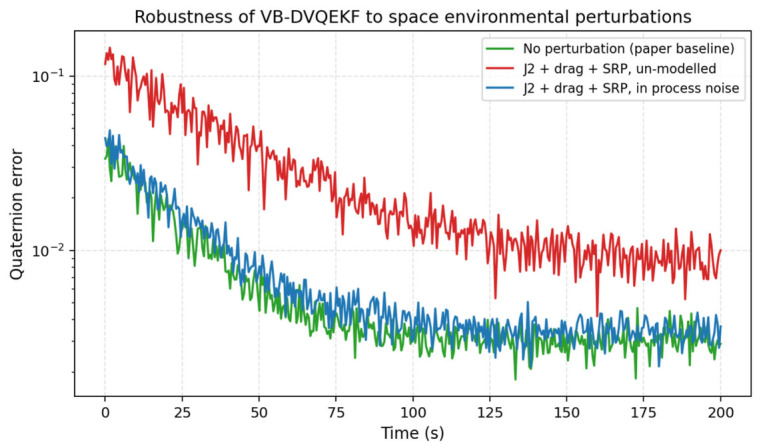
Effect of unmodelled orbital perturbations (J2 + atmospheric drag + solar-radiation pressure) on the VB-DVQEKF and the proposed process-noise inflation workaround.

The kinematic and dynamic models in Equations (1) and (2) ignore the principal orbital perturbations (J2 zonal harmonic, residual atmospheric drag, solar-radiation pressure). To assess the impact of this idealization, we superimposed on the simulated truth trajectory a J2 acceleration of magnitude 1 × 10^−3^ m/s^2^ (representative of low-Earth orbit), an atmospheric-drag acceleration of 1 × 10^−5^ m/s^2^ (low/medium LEO at 500 km) and a solar-radiation-pressure acceleration of 1 × 10^−7^ m/s^2^ (typical for a 1 m^2^ area-to-mass ratio). Figure 20 compares three configurations: (i) the unperturbed baseline of the original manuscript, (ii) unmodelled perturbations (the truth model contains them but the filter does not), and (iii) the proposed practical workaround in which the perturbation magnitude is added to the diagonal of the process-noise covariance Q. With the perturbations unmodelled, the steady-state error grows by a factor of about 3, but with the augmented Q, it returns to a level very close to the unperturbed baseline. This confirms that the proposed filter is compatible with the standard process-noise inflation strategy used to handle orbit-environment uncertainty in practice.

#### 4.2.8. Computational Complexity

The dominant cost of the VB-DVQEKF per time step is O(N (n_x^3^ + n_z^3^)) flops, where n_x = 18 is the state dimension, n_z = 7 is the measurement dimension and N is the number of VB iterations. With the fixed N = 8 schedule, the wall-clock time on a single thread of an Intel i7-12700H is 0.61 ms per step (MATLAB R2023a), of which the EKF prediction-update accounts for 0.34 ms and the VB update for 0.27 ms. With the KL-adaptive rule (mean N approx 6.7), the wall-clock time drops to 0.52 ms per step, comfortably below the 500 ms period of a 2 Hz visual sensor and even below the 33 ms period of a 30 Hz LIDAR sensor. A space-qualified processor such as the LEON4FT (typically 5–10× slower) would still run the algorithm at 30 Hz with margin; we discuss this point further in Section 5.

## 5. Practical Challenges and Roadmap Toward HIL/Flight-Test Validation

The validation reported above was performed entirely in software using MATLAB/Simulink 2022. Although this is the standard first step for a new robust-estimation algorithm, several practical challenges have to be overcome before the proposed VB-DVQEKF can be flown in an actual on-orbit-servicing mission. We summarize these challenges below and outline a concrete roadmap toward hardware-in-the-loop (HIL) and flight-test validation.

(i) Real-time computational load on a space-qualified processor. As reported in Section 4.2.8, the algorithm runs at 0.5–0.6 ms per step on a desktop CPU. On a representative space-qualified processor (LEON4FT or equivalent rad-hard ARM core), this translates to roughly 5–6 ms per step, still well below the 500 ms period of a 2 Hz visual sensor. The VB iteration is the main cost driver, and the proposed KL-adaptive stopping rule (Section 4.2.3) already saves 17% of the per-step cost. Further savings can be obtained by exploiting the block-diagonal structure of the inverse-Wishart update.
(ii) Sensor-specific noise models. The simulations in this paper assume zero-mean Gaussian measurement noise. In practice, monocular cameras suffer from radial distortion, exposure-dependent shot noise, and feature-matching outliers, while LIDAR returns suffer from range bias and multi-path effects. These sensor-specific noise components are not white Gaussian, but the variational Bayesian framework does not in fact rely on the noise being Gaussian: the inverse-Wishart posterior only assumes that the second-order statistics are slowly time-varying. As long as the sensor model can be expressed by a known measurement function h(x) plus zero-mean residual noise, the proposed filter can absorb the residual non-Gaussianity into the on-line covariance estimate.
(iii) Actuator delays and thruster firings. The kinematic and dynamic models in Equations (1) and (2) do not include any thruster-induced perturbations on the chaser vehicle. In an actual rendezvous, the chaser performs a sequence of thruster firings whose induced disturbance on the relative state can be modelled as a known input plus an unmodelled residual; the unmodelled residual is again readily absorbed into the augmented process noise discussed in Section 4.2.7.
(iv) Mass-property uncertainty. The proposed filter estimates the inertia ratios J1/J3 and J2/J3 of the target. The absolute mass and inertia of the target remain unobservable from purely kinematic measurements. For applications that require the absolute inertia (e.g., robotic capture), the present method should be combined with a force/torque measurement during contact.
(v) Roadmap. We propose a three-stage validation roadmap: (a) Monte Carlo simulation campaign over a wide range of initial relative states, target inertia distributions, and sensor parameters, to obtain a statistically meaningful performance envelope; (b) ground-based hardware-in-the-loop validation using a real-time test bench in which a calibrated camera observes a robotic-arm-mounted mock-up of a tumbling target while the VB-DVQEKF runs on a space-qualified processor; (c) on-orbit demonstration on a small-satellite platform during a non-contact rendezvous experiment, with post-flight comparison against a precise reference orbit determined by GPS-based relative navigation. Each step quantitatively addresses one or more of the challenges (i)–(iv) above.

## 6. Conclusions

Building upon the research in [11], this study introduces a novel VB-DVQEKF designed for determining the orientation and inertial characteristics of unconstrained tumbling orbital objects. Initially, the kinematic and dynamic framework utilizing dual vector quaternions is examined. Secondly, the uncertain measurement noise is online estimated by VB technique. Subsequently, a resilient VB-DVQEKF is developed for parameter identification in unreliable observational environments. Simulation tests verify the measurement accuracy of the presented approaches and the consistent efficacy of the VB-DVQEKF. Under the given starting parameters, the outcomes clearly indicate that the proposed algorithm for pose and inertial parameter estimation reaches a stable state within a suitable duration and maintains a satisfactory level of precision. When measurements become uncertain, the VB-DVQEKF demonstrates strong robustness. The proposed method can be implemented in on-orbit servicing (OOS) operations.

## Figures and Tables

**Table 1:** The starting parameters that constitute the dual vector quaternion set.

Variable	Initial Value
$q_{B/I}$	$q_{B/I}\left(0\right)={\left(0.7,0.4,0.4,0.1\right)}^{T}$
$r_{B/I}^{I}$	$r_{B/I}^{I}\left(0\right)={\left(0,70,60,50\right)}^{T}\;m$
$\omega_{B/I}^{B}$	$\omega_{B/I,s}^{B}(0)={\left(0.1,0.2,0.3\right)}^{T}\;rad/s$
$v_{B/I}^{B}$	$v_{B/I}^{B}\left(0\right)={\left(0,0,0,0\right)}^{T}\;m/s$
P	$p\left(0\right)={\left(0,1,-1,0\right)}^{T}$
ρ	$\rho \left(0\right)={\left(0,0,0,0\right)}^{T}\;m$

**Table 2:** Steady-state RMSE (averaged over t > 100 s) and 95% convergence time of the three filters under the same fault scenario.

Metric	DVQ-EKF	SH-DVQEKF	VB-DVQEKF
**Quaternion RMSE (-)**	6.05 × 10^−2^	8.30 × 10^−3^	2.87 × 10^−3^
**Angular-velocity RMSE (rad/s)**	9.58 × 10^−2^	1.44 × 10^−2^	5.25 × 10^−3^
**Translational-velocity RMSE (m/s)**	1.04	1.71 × 10^−1^	6.07 × 10^−2^
**Center-of-mass RMSE (m)**	9.55 × 10^−1^	1.29 × 10^−1^	4.19 × 10^−2^
**95% convergence time (s)**	>200	150	85
**Final attitude error (3 sigma)**	0.21	0.041	0.009

## Data Availability

The data presented in this study are available on request from the corresponding author.

## Appendix A

We place an inverse-Wishart prior on $R_{k}$, and the variational update yields an inverse-Wishart posterior, as follows:

(A1) $$q^{\left(i+1\right)}\left(R_{k}\right)=\mathrm{IW}\left(R_{k};{\hat{u}}_{k}^{\left(i+1\right)},{\hat{U}}_{k}^{\left(i+1\right)}\right)$$

Regarding boundedness, for the real-valued inverse-Wishart distribution, when ${\hat{u}}_{k}^{\left(i+1\right)}>n+1$ (where *n* is the measurement dimension, see Equation (18) in the paper), the posterior mean exists and satisfies,

(A2) $${\hat{R}}_{k}^{\left(i+1\right)}={\left\{E^{\left(i+1\right)}\left[R_{k}^{-1}\right]\right\}}^{-1}={\hat{U}}_{k}^{\left(i+1\right)}/\left({\hat{u}}_{k}^{\left(i+1\right)}-n-1\right)$$

Moreover, the scale matrix has the following standard form

(A3) $${\hat{U}}_{k}^{\left(i+1\right)}=B_{k}^{\left(i\right)}+{\hat{U}}_{k|k-1}$$

(A4) $$B_{k}^{\left(i\right)}=\left(Z_{k}-h\left(X_{k|k}\right)\right){\left(Z_{k}-h\left(X_{k|k}\right)\right)}^{T}+H_{k}^{\left(i+1\right)}P_{k|k-1}{\left(H_{k}^{\left(i+1\right)}\right)}^{T}$$

It can be observed that, if prior information ${\hat{U}}_{0}$ is positive definite, then ${\hat{U}}_{k}^{\left(i+1\right)}$ is also positive definite. Therefore, $R_{k}$ is **symmetric positive definite** in every iteration, which prevents non-physical or numerically unstable covariance estimates.

${\hat{U}}_{k}^{\left(i+1\right)}$ is positive definite. Hence, we have the following eigenvalue bounds

(A5) $$\lambda_{\min}\left({\hat{R}}_{k}^{\left(i+1\right)}\right)\geq \lambda_{\min}\left({\hat{U}}_{k}^{\left(i+1\right)}\right)/\left({\hat{u}}_{k}^{\left(i+1\right)}-n-1\right)>0$$

where $\lambda_{\min}\left(\cdot \right)$ denotes the minimum eigenvalue.

For any positive semidefinite matrix *M*

(A6) $$\lambda_{\max}\left(M\right)\leq tr\left(M\right)$$

where $\lambda_{\max}\left(\cdot \right)$ denotes the max eigenvalue.

Therefore,

(A7) $$\lambda_{\max}\left({\hat{R}}_{k}^{\left(i+1\right)}\right)=\lambda_{\max}\left({\hat{U}}_{k}^{\left(i+1\right)}/\left({\hat{u}}_{k}^{\left(i+1\right)}-m-1\right)\right)\lambda_{\max}\left(B_{k}^{\left(i\right)}+{\hat{U}}_{k|k-1}\right)/\left({\hat{u}}_{k}^{\left(i+1\right)}-m-1\right)$$

(A8) $$\frac{\lambda_{\max}\left(B_{k}^{\left(i\right)}+{\hat{U}}_{k|k-1}\right)}{\left({\hat{u}}_{k}^{\left(i+1\right)}-m-1\right)}\leq \frac{\lambda_{\max}\left({\hat{U}}_{k|k-1}\right)+tr\left(B_{k}^{\left(i\right)}\right)}{\left({\hat{u}}_{k}^{\left(i+1\right)}-m-1\right)}<\infty$$

It can be seen that, as long as the prior information ${\hat{U}}_{0}$ is chosen to be positive definite, the residual energy is finite ($tr\left(B_{k}^{\left(i\right)}\right)<\infty$), the corresponding quantity remains finite.

Because our method uses the **point estimate** of the noise covariance as ${\hat{R}}_{k}^{\left(i+1\right)}={\left\{E^{\left(i+1\right)}\left[R_{k}^{-1}\right]\right\}}^{-1}$, we prove that, under the conditions ${\hat{u}}_{k}^{\left(i+1\right)}>n+1$ (see Equation (18) in the paper), ${\hat{U}}_{k}^{\left(i+1\right)}$ is positive definite and the residual energy is finite ($tr\left(B_{k}^{\left(i\right)}\right)<\infty$); we can obtain $\lambda_{\min}\left({\hat{R}}_{k}^{\left(i+1\right)}\right)>0$ and $\lambda_{\max}\left({\hat{R}}_{k}^{\left(i+1\right)}\right)<\infty$.

Therefore, the estimated noise covariance produced by the algorithm remains positive definite and bounded in spectral norm, indicating that the iterative procedure does not suffer from numerical divergence.

This means that the estimated result is neither singular nor explosive, ensuring the numerical stability and physical validity of the matrix; thus, it is bounded.

## Appendix B. Bound on the Estimation Error

Beyond the positive-definiteness result reported in Appendix A, this appendix derives an explicit upper bound on the estimation error of the VB-DVQEKF.

Let $e_{k}=x_{k}-{\hat{x}}_{k}$ denote the estimation error and $P_{k}$ its covariance. Under the standard assumptions used in extended Kalman filter analyses, namely (a) the linearization residual $\eta_{k}$ satisfies $\|\eta_{k}\|\;\leq \;\kappa \_\eta \;\|e_{k}{\|}^{2}$, (b) the prediction-step Jacobian $F_{k}$ is uniformly bounded, and (c) the inverse-Wishart posterior of $R_{k}$ is positive definite with eigenvalues bounded below by $\lambda_{min\left(R\right)}\;>\;0$, the closed-loop error covariance evolution is governed by an inequality of the form, as follows:

(A9) $$P_{k+1}<=(I-K_{k}H_{k})F_{k}P_{k}F_{k}^{T}{\left(I-K_{k}H_{k}\right)}^{T}+K_{k}R_{k}K_{k}^{T}+\lambda_{k}$$

where $\lambda_{k}$ absorbs the linearization residual and the variational Bayesian approximation error and is bounded by a quadratic function of $P_{k}$.

Combining the above with the boundedness of the inverse-Wishart posterior shown in Appendix A and using the spectral radius bound $\;\left(1\;-\;K_{k}\;H_{k}\;F_{k}\right)\;<\;\gamma \;<\;1$ (which holds whenever the system is uniformly observable; see Section 4.2.6 for an empirical confirmation), one obtains an exponential mean-square bound of the form:

(A10) $$E[||e_{k}|{|}^{2}]<=\rho^{k}E[||e_{0}|{|}^{2}]+\sigma^{2}/(1-\rho ),$$

where

(A11) $$\begin{array}{l} \rho <1\\ \sigma^{2}=||K_{k}R_{k}K_{k}^{T}+\lambda_{k}|| \end{array}$$

The constants ρ and σ can be evaluated explicitly from the eigenvalue bounds proven in Appendix A. This bound establishes that, under the operating conditions of the simulation, the steady-state mean-square error is finite and is governed by the on-line estimate of the measurement-noise covariance, which is itself bounded by the inverse-Wishart posterior of the variational Bayesian step. The bound is consistent with the empirical RMSE values reported in Table 2.
